# Epithelial to Mesenchymal Transition Regulates Surface PD-L1 via CMTM6 and CMTM7 Induction in Breast Cancer

**DOI:** 10.3390/cancers13051165

**Published:** 2021-03-09

**Authors:** Malina Xiao, Meriem Hasmim, Audrey Lequeux, Kris Van Moer, Tuan Zea Tan, Christine Gilles, Brett G. Hollier, Jean Paul Thiery, Guy Berchem, Bassam Janji, Muhammad Zaeem Noman

**Affiliations:** 1 Tumor Immunotherapy and Microenvironment (TIME) Group, Department of Oncology, Luxembourg Institute of Health (LIH), Luxembourg City, L-1526 Strassen, Luxembourg; malina.xiao@lih.lu (M.X.); meriem.hasmim@lih.lu (M.H.); audrey.lequeux@lih.lu (A.L.); kris.vanmoer@lih.lu (K.V.M.); berchem.guy@chl.lu (G.B.); 2 Cancer Science Institute of Singapore, National University of Singapore, Singapore 117599, Singapore; csittz@nus.edu.sg; 3 GIGA-Cancer, Laboratory of Tumor and Development Biology, University of Liège, 4000 Liège, Belgium; cgilles@ulg.ac.be; 4 Australian Prostate Cancer Research Centre-Queensland (APCRC-Q), School of Biomedical Sciences, Faculty of Health, Queensland University of Technology, Princess Alexandra Hospital, Translational Research Institute, Brisbane, QLD 4102, Australia; b.hollier@qut.edu.au; 5 Bioland Laboratory, Guangzhou Regenerative Medicine and Health, Guangdong Laboratory, Guangzhou 510320, China; tjp@nus.edu.sg; 6 Department of Hemato-Oncology, Centre Hospitalier du Luxembourg, L-1210 Luxembourg, Luxembourg

**Keywords:** breast cancer, epithelial to mesenchymal transition, snail1, CMTM family, immune checkpoints

## Abstract

**Simple Summary:**

Epithelial to mesenchymal transition (EMT) is a mechanism endowing tumor cells with aggressive properties and escape immune surveillance. Here, we reported that activating EMT process in breast cancer cells upregulates CMTM6, an essential protein required for cell surface expression of PD-L1. In addition to CMTM6, our in silico data on triple negative breast cancer patients showed a positive correlation between EMT markers and two other members of CMTM family (CMTM3 and CMTM7). These results were validated in EMT-inducible MCF-7 model that exhibited a downregulation of cell surface expression of PD-L1 by dual targeting CMTM6 and CMTM7. Considering the prominent role of PD-L1 in tumor immune evasion, our study provides an additional clue on how PD-L1 is regulated in aggressive breast cancer cells and pave the way to assess the therapeutic benefit of EMT inhibitors in combination with anti PD-L1 blockade in highly aggressive breast cancer patients.

**Abstract:**

CMTM6 is a critical regulator of cell surface expression of PD-L1 in tumor cells, but little is known about the transcriptional regulation of CMTM6. Here we report that the expression of CMTM6 positively correlates with the epithelial to mesenchymal transition (EMT) score in breast cancer cell lines and with the major EMT marker Vimentin in triple-negative breast cancers (TNBC). We showed that CMTM6 is concomitantly overexpressed with PD-L1 in breast mesenchymal compared with the epithelial cells. Driving a mesenchymal phenotype in SNAI1-inducible MCF-7 cells (MCF-7^Mes^ cells) increased both PD-L1 and CMTM6. CMTM6 silencing in MCF-7^Mes^ cells partially reduced cell surface expression of PD-L1, indicating that a proportion of the PD-L1 on the surface of MCF-7^Mes^ cells depends on CMTM6. We also found a positive correlation between CMTM3 and CMTM7 expression with EMT score in breast cancer cells, and with Vimentin in TNBC patients. Dual knockdown of CMTM6 and CMTM7 significantly decreased PD-L1 surface expression in MCF-7^Mes^ cells, indicating that both CMTM6 and CMTM7 regulate the expression of PD-L1. This study highlights the importance of CMTM6 and CMTM7 in EMT-induced PD-L1 and suggests that EMT, CMTM6 or CMTM7 modulators can be combined with anti-PD-L1 in patients with highly aggressive breast cancer.

## 1. Introduction

Epithelial to mesenchymal transition (EMT) is a dynamic and reversible process, whereby epithelial cells switch their phenotype into a more motile and invasive mesenchymal phenotype. EMT was first described as a physiological program occurring during embryogenesis. Subsequently, EMT was proposed as a key mechanism involved in carcinoma progression and malignancy [1]. EMT is orchestrated by EMT-inducing transcription factors (EMT-TFs), including SNAIL, ZEB, and TWIST families [2,3]. The involvement of EMT-TFs in pro-invasive, metastatic, and drug resistance has been well-documented in tumor cells [4]. Over the last decade, evidence has highlighted the role of EMT-TFs in immune escape [5]. Also, we have previously reported that the EMT phenotype in human breast cancer cells is associated with an inhibition of CTL-mediated tumor cell lysis [6,7]. Furthermore, we and others have reported that EMT-TFs such as SNAI1 or ZEB1 bind directly to the proximal promoter of CD47 and PD-L1 and subsequently induced their expression in breast and lung cancer cells [8,9,10]. Indeed, we showed that macrophage-mediated phagocytosis was activated by either blocking the EMT-dependent CD47 overexpression via anti-CD47 antibody or silencing of SNAI1 or ZEB1 via siRNA in mesenchymal MDA-MB-231 breast cancer cells [10]. Furthermore, the anti-PD-L1 antibody reduced tumor growth, lung metastasis, and exhausted CD8+ T cells in a mouse lung carcinoma model [8]. However, whether EMT-TFs could also be involved in post-translational regulation of immune checkpoints remains unclear.

Proteins containing the CKLF-like MARVEL transmembrane (CMTM) domain have been involved in the regulation of the trafficking of transmembrane and secretory proteins [11]. The CMTM family contains eight members (CMTM1–8) located in three distinct gene clusters on chromosome 16 (CMTM1-4), chromosome 14 (CMTM5), and chromosome 3 (CMTM6-8) [12]. Recently, the uncharacterized protein CMTM6 has been identified as a critical regulator of PD-L1 surface expression in several types of cancer cells, including breast cancer cells. CMTM6 has been reported to be essential for efficient recycling and cell surface stabilization of PD-L1 [13]. Based on our previous findings showing the involvement of EMT-TFs in the upregulation of PD-L1, we investigated whether EMT-dependent increase of PD-L1 on the surface of breast cancer cells involves CMTM6.

In the current study, we demonstrated that upon SNAI1-dependent induction of EMT, the expression of CMTM6 protein was significantly increased. CMTM6 silencing in mesenchymal breast cancer cells partially decreased PD-L1 expression on their cell surface, while dual targeting of CMTM6 and CMTM7 significantly decreased PD-L1 in these cells. The translational aspect of our study is underscored by data showing a positive correlation between CMTM6, CMTM7, and the major mesenchymal marker Vimentin in TNBC patients. 

## 2. Materials and Methods

### 2.1. Cell Lines

Human breast cancer cell lines MCF-7 and MDA-MB-231 were purchased from ATCC (American Type Culture Collection, Manassas, VA, USA). MCF-7 iSNAI1 cells were generated by cloning Snail cDNA into the doxycycline-inducible pINDUCER20 lentiviral expression construct [14]. Cell lines were mycoplasma-free according to MycoAlert assay (Lonza, Basel, Switzerland) and were maintained in DMEM supplemented with 10% Fetal Bovine Serum and 1% penicillin/streptomycin at 37 °C and 5% CO_2_.

### 2.2. SNAI1 Induction

MCF7 iSNAI1 cells were seeded 24 h prior to doxycycline treatment (Sigma-Aldrich, St. Louis, MO, USA) and treated at the indicated concentrations and time-points. Medium containing doxycycline was refreshed every 48 h. SNAI1 overexpression was confirmed at mRNA and protein levels.

### 2.3. siRNA Transfection

MDA-MB-231 and MCF-7 iSNAI1 cells were seeded in a 6-well plate and transfected using Lipofectamine RNAiMax, according to the manufacturer’s recommendations. Prior to transfection, a mix of Lipofectamine and siRNA in Opti-MEM medium was prepared and incubated for 10 min at room temperature (RT). Cells were incubated for 4 h to 6 h with the mix at 37 °C and 5% CO_2_. A complete medium was next added to cells for prolonged incubation. Finally, cells were harvested 48 h post-transfection for RNA and protein analyses.

### 2.4. RNA Extraction, First-Strand cDNA Synthesis, and SYBR Green RT-qPCR

Total RNAs were extracted using the Nucleospin RNA Plus Kit (Macherey-Nagel, Dueren, Germany). RNA was reverse-transcribed using the Maxima First-Strand cDNA Synthesis Kit (Thermofisher Scientific, Schwerte, Germany) and amplified by qPCR using the Power SYBR Green PCR Master Mix (Eurogentec, Seraing, Belgium). mRNA levels were normalized to ACTIN mRNA levels. Primers are listed in the key resources table (Supplementary Materials Table S1).

### 2.5. Immunoblot Assay

Cells were lysed in a lysis buffer containing 10% SDS, glycerol, tris-HCl pH 6.8 1M, halt protease and phosphatase inhibitor cocktail. Protein samples were loaded, separated, and transferred to nitrocellulose Western blot membranes (VWR). Membranes were blocked with 5% milk in TBS-T. Primary antibodies were incubated with membranes overnight at 4 °C in 5% BSA TBS-T. Secondary antibodies were incubated in 5% milk with TBS-T for 1 h at RT. After washing, staining was revealed with Western Lightning Ultra mainly on ImageQuant LAS4000 (GE Healthcare Life Sciences, Machelen, Belgium) or X-ray film depending on the antibody’s sensitivity. Primary antibodies included E-cadherin (Cell signaling, #3195S), N-cadherin (Cell signaling, #13116S), Vimentin (Cell signaling, #5741S), Snail (Cell signaling, #3879S), ZEB1 (Cell signaling, #3396S), PD-L1 (Cell signaling, #13684S) and CMTM6 (Sigma-Aldrich, HPA026980).

### 2.6. Immunofluorescence Microscopy

MDA-MB-231 and MCF-7 iSNAI1 cells were seeded in an 8-well IBIDI chamber. Cells were fixed for 20 min at RT in 4% PFA and permeabilized with 0.25% Tween-20 in PBS for 10 min at RT. Cells were next blocked with 5% BSA in 0.25% Tween-20 in PBS for 1 h at RT. Cells were incubated with primary antibodies in 0.1% BSA for 2 h at RT (E-cadherin 1/200, CMTM6 1/1000), and washed twice with PBS. The fluorescent conjugated secondary antibodies were incubated at a 1/400 dilution ratio in 0.1% BSA for 1 h at RT or with Actin-Stain 488 Phalloidin at 1/400 dilution ratio and washed twice with PBS. Finally, cells were stained with DAPI for 5 min at RT and washed twice with PBS. Images were acquired on confocal LSM880 Airy (Carl Zeiss), and scale bars were added with the ZEN software. The antibodies used included CMTM6 (Sigma-Aldrich, HPA026980), Acti-Stain 488 Phalloidin (Cytoskeleton, Inc., Denver, CO, USA, #PHDG1-A) and Goat anti-Rabbit IgG Secondary Antibody, Alexa Fluor Plus 488 (Thermofischer Scientific, #A32731).

### 2.7. Flow Cytometry

Cells were detached with 10 mM EDTA for 10 min at 37 °C, washed with PBS and centrifuged at 400× *g* for 5 min at 4 °C. Cells were next incubated with fluorochrome-conjugated antibodies in the dark for 30 min at 4 °C. Cells were washed twice in MACS buffer (Miltenyi, Bergisch Gladbach, Germany) at 400× *g* for 5 min at 4 °C. Samples were processed on CytoFLEX flow cytometry (Beckman Coulter, Brea, CA, USA), and data were analyzed by using FlowJo software. The following antibodies were used: CD274 (Thermofisher Scientific, #12-5983-42), Mouse IgG1 kappa Isotype Control (Thermofisher Scientific, #12-4714-82) and LIVE/DEAD™ Fixable Near-IR Dead Cell Stain Kit (Thermofisher Scientific, L34976).

### 2.8. In Silico Correlation Analysis of CMTM Family with EMT Score in Breast Cancer Cell Lines Reported in the CCLE Database and with Vimentin in the METABRIC

For breast cancer cell lines, the EMT score was computed using a previously defined EMT signature and the two-sample Kolmogorov-Smirnov-based method [15]. A breast cancer patient from METABRIC cohort [16,17] was downloaded from cbioportal v3.4.12. TNBC patients were defined according to their ER-, PR-, and HER2- status. The mRNA expression z-score of VIM and all members of the CMTM family (CMTM1 to 8) was defined at ±0.5 z-score threshold for all genes in 299 TNBC patients. The mRNA co-expression of VIM and CMTM members was defined on cBioPortal (https://www.cbioportal.org/) (accessed on 2 January 2021). Correlation analyses were performed by Spearman test in breast cancer cell lines and the METABRIC database.

### 2.9. Quantification and Statistical Analysis

Western blot densitometry quantification was performed using ImageJ software. Statistical analyses were done using unpaired two-tailed Student’s *t*-test in GraphPad Prism version 8 (GraphPad Software, La Jolla, CA, USA). Experimental data are represented as the mean ± SEM. A *p*-value of less than 0.05 was considered significant. * *p* < 0.05; ** *p* < 0.01; *** *p* < 0.001; ns: not significant. The Spearman correlation coefficient was used to assess correlation by Matlab^®^ R2016b version 9.1.0.960167 (MathWorks; Natick, MA, USA). *In silico* correlation analysis between CMTM family and EMT score was performed using the Spearman test.

## 3. Results and Discussion

### 3.1. CMTM6 Is Upregulated in Mesenchymal as Compared with Epithelial Breast Cancer Cells and Regulates PD-L1 Cell Surface Expression

**Figure 1:**
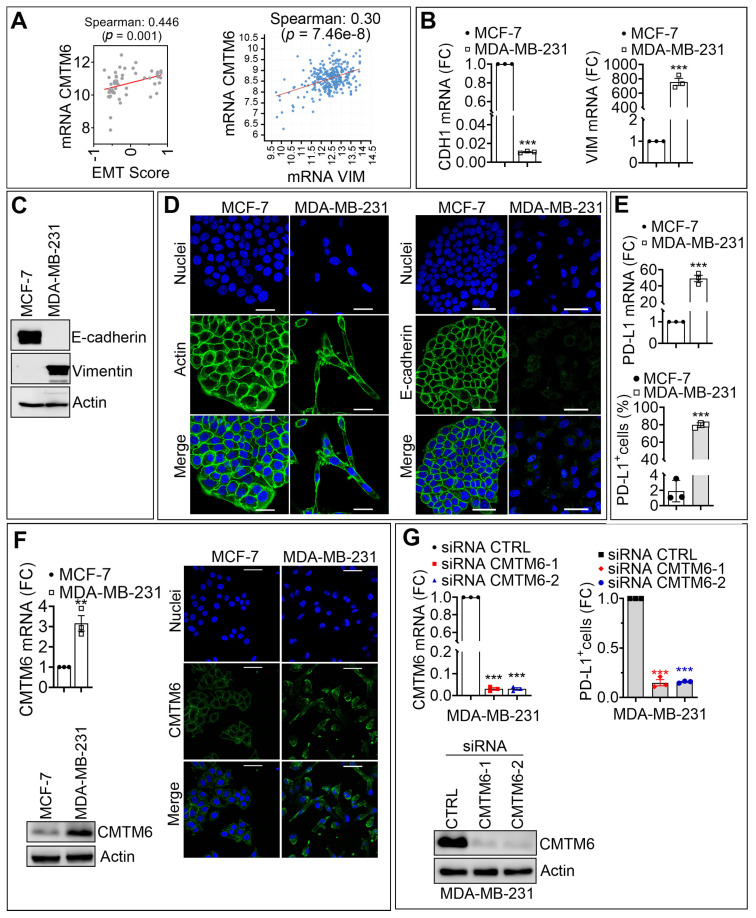
CMTM6 is upregulated in mesenchymal as compared with epithelial breast cancer cells and regulates PD-L1 cell surface expression. (**A**) Left panel: Spearman correlation between the EMT score and the mRNA expression of CMTM6 in 51 breast cancer cell lines. Right panel: Spearman correlation between Vimentin (VIM) and CMTM6 mRNA in 299 TNBC patients. The corresponding *p*-values are reported. (**B**,**C**) Comparative expression of mRNA (**B**) and protein (**C**) of E-cadherin and Vimentin in MCF-7 and MDA-MB-231 cells. Results in B are reported as fold change (FC) relative to MCF-7 cells. β-Actin was used as a loading control in C. (**D**) Representative immunofluorescence images of Actin or E-cadherin (green) in MCF-7 and MDA-MB-231 cells. Nuclei are stained with DAPI (blue). Scale bar: 50 µm. (**E**) mRNA expression (upper panel) and surface expression (lower panel) of PD-L1 in MCF-7 and MDA-MB-231 cells. The results in the upper panel are reported as FC relative to MCF-7 cells, and the results in the lower panel are reported as a percentage (%) of cells expressing PD-L1. (**F**) Expression of CMTM6 mRNA (upper left panel) and protein (lower left panel) in MCF-7 and MDA-MB-231 cells. Results of the upper left panel are reported as FC relative to MCF-7 cells. β-Actin is used as a loading control in the lower-left panel. Right panels: representative immunofluorescence images of CMTM6 (green) in MCF-7 and MDA-MB-231 cells. Nuclei are stained with DAPI (blue). Scale bar: 50 µm. (**G**) Expression of CMTM6 mRNA (upper left panel) and protein (lower left panel) in MDA-MB-231 cells transfected with control siRNA (CTRL siRNA) or two different CMTM6 siRNAs (siCMTM6-1 and -2). Results in the upper left panel are reported as FC relative to MCF-7 cells. β-Actin is used as a loading control in the lower left panel. Right panel: Flow cytometry quantification of surface PD-L1 (PD-L1+ cells) as previously. Results are reported as FC relative to CTRL siRNA cells. Results in (**B**,**E**,**F**) (upper left panel), and **G** (upper panels) represent the average of three independent experiments. Statistically significant differences (indicated by asterisks) are calculated compared with control conditions using an unpaired two-tailed Student’s *t*-test (** *p* < 0.01 and *** *p* < 0.001). For all panels, each dot represents one individual experiment.

We first investigated the relationship between EMT score and the expression of CMTM6 in a broad range of breast cancer cells. The EMT score was previously established based on the expression of genes involved in the EMT process, such as SNAI1, ZEB1, CDH1, and VIMENTIN (VIM). Based on their transcriptional profile, this score allows the classification of samples according to their epithelial (negative EMT score) or mesenchymal (positive EMT score) phenotype [15]. Our *in silico* analysis showed a highly significant positive correlation between CMTM6 expression and EMT score in breast cancer cell lines described in the CCLE database (Figure 1A, left panel). Accordingly, we showed that the expression of CMTM6 was positively correlated with the expression of the major EMT marker VIM in TNBC of the METABRIC cohort described in the TCGA database (Figure 1A, right panel). Based on these in silico data, we selected MCF-7 and MDA-MB-231 breast cancer cell lines as representative epithelial and mesenchymal cell lines, respectively, according to their EMT score. MCF-7 cells are characterized as a luminal A breast cancer subtype and harbor wild type BRCA1. MDA-MB-231 cells are reported as triple-negative breast cancer (TNBC) subtype and also harbor wild-type BRCA1 [18].

We evaluated the mRNA and protein expression of epithelial and mesenchymal markers in both cell lines. As expected, mRNA and protein expression level of CDH1 was higher in epithelial MCF-7 cells relative to mesenchymal MDA-MB-231 cells, whereas mRNA and protein expression levels of VIM were significantly higher in mesenchymal MDA-MB-231 cells relative to epithelial MCF-7 cells (Figure 1B,C). These results were further confirmed by confocal microscopy, showing a decrease in the expression of E-cadherin in MDA-MB-231 along with a spindle-shaped morphology as compared with MCF-7, which exhibited a typical cobblestone phenotype (Figure 1D). Next, we assessed whether there is a correlation between the expression of PD-L1 and the expression of EMT markers and CMTM6 in mesenchymal MDA-MB-231 and epithelial MCF-7 cells. Interestingly, we found that both mRNA and cell surface expression levels of PD-L1 were significantly upregulated (more than 40-fold for the mRNA and 80-fold for the protein) in mesenchymal MDA-MB-231 cells displaying high expression level of CMTM6 (both mRNA and protein) as compared with epithelial MCF-7 cells displaying low expression level of CMTM6 (Figure 1E,F). Supplementary Materials Figure S1, showed a densitometry quantification of CMTM6 protein of five individual experiments performed on MDA-MB-231 and MCF-7 cells. Together, our in vitro results confirm the *in silico* data highlighting the positive correlation between EMT and CMTM6 expression in breast cancers. As CMTM6 was reported to play a crucial role in the stabilization of PD-L1 on the cell surface [13], we, therefore, assessed to what extent the upregulation of PD-L1, observed in mesenchymal MDA-MB-231 cells, depends on the expression of CMTM6. To address this issue, mesenchymal MDA-MB-231 cells were transfected with two different CMTM6 siRNA sequences. Figure 1G (left panels) shows the efficiency of the siRNA to inhibit mRNA and protein expression of CMTM6. Strikingly, CMTM6 silencing in mesenchymal MDA-MB-231 cells strongly reduced PD-L1 surface expression (80–90% inhibition) relative to PD-L1 surface expression in CTRL siRNA—transfected cells (Figure 1G, right panel). Supplementary Materials Figure S2 shows densitometry quantification of CMTM6 protein from four individual experiments performed in MDA-MB-231 cells transfected with control or CMTM6 siRNAs. Our results establish direct evidence that the overexpression of PD-L1 on the surface of mesenchymal breast tumor cells is most likely dependent on CMTM6. Our data provide an additional clue on the involvement of the EMT process in the regulation of CMTM6.

### 3.2. SNAI1-Dependent Increase in CMTM6 Protein Contributes to PD-L1 Cell Surface Expression in Breast Cancer Cells

**Figure 2:**
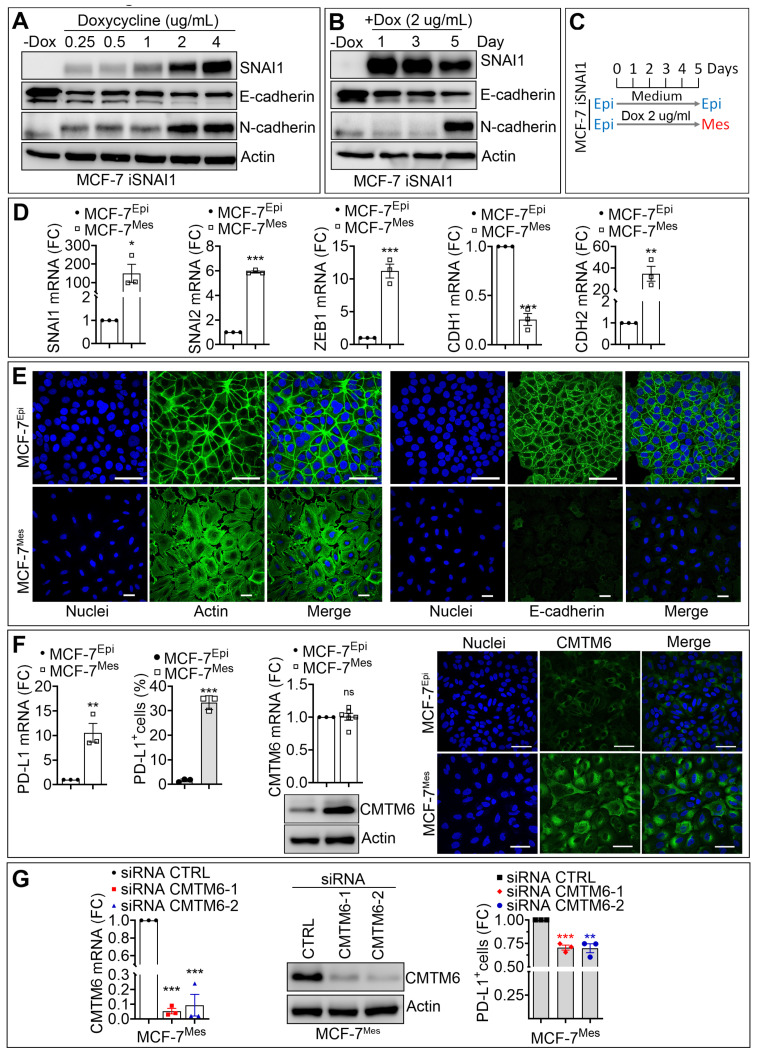
SNAI1-dependent increase in CMTM6 contributes to PD-L1 cell surface expression in breast cancer cells. (**A**,**B**) The expression of SNAI1, E-cadherin (E-CAD), N-cadherin (N-CAD) protein in MCF7 iSNAI1 cells untreated (-DOX) or treated with different doses (0.25, 0.5, 1, 2, 4 µg/mL) of doxycycline for 5 days (**A**) and at 2 µg/mL at different time points (1, 3, and 5 days) (**B**) β-Actin was used as a loading control. (**C**) In vitro doxycycline treatment strategy of MCF-7 iSNAI1 cells. Mes cells were treated with 2 µg/mL of DOX at day 0, 2, and 4 and harvested at day 5 for analysis. At day 5, DOX-treated cells switched to a mesenchymal phenotype (MCF-7^Mes^) while untreated cells kept their epithelial phenotype (MCF-7^Epi^). (**D**) The expression of SNAI1, SNAI2, ZEB1, CDH1, and CDH2 mRNA by RT-qPCR in MCF-7^Epi^ and MCF-7^Mes^ cells. Results are reported as FC relative to MCF-7^Epi^ cells. (**E**) Representative immunofluorescence images of Actin and E-cadherin (green) in MCF-7^Epi^ and MCF-7^Mes^ cells. Nuclei are stained with DAPI (blue). Scale bar: 50 µm. (**F**) Expression of PD-L1 mRNA (left panel), PD-L1 cell surface (middle panel), and CMTM6 mRNA and protein (right panels) in MCF-7^Epi^ and MCF-7^Mes^ cells. Scale bar: 50 µm. (**G**) Expression of CMTM6 mRNA (left) and CMTM6 protein (middle) in MCF-7^Mes^ cells transfected with CTRL siRNA or two different CMTM6-targeting siRNAs (siCMTM6-1 and -2). Right panel: Flow cytometry quantification of PD-L1+ cells transfected with two different CMTM6-targeting siRNAs (siCMTM6-1 and -2). Results in left and right panels are reported as FC relative to control cells. For all panels, each dot represents one independent experiment and results represent the average all independent experiments. Statistically significant differences (indicated by asterisks) are calculated compared with control conditions using an unpaired two-tailed Student’s *t*-test (* *p* < 0.05; ** *p* < 0.01 and *** *p* < 0.001).

The critical role of EMT-TFs in cancer aggressiveness and immune escape is well established. In pancreatic cancers, ZEB1 has been described as a major factor in driving metastasis, stemness, and colonization capacity [19]. In breast cancer, SNAI1, but not SNAI2, has been reported to play an important role in primary tumor growth and metastasis. Indeed, SNAI1 binds to the promoter of ZEB1 and induces its expression in MDA-MB-231 cells [20]. We, therefore, focused on SNAI1 to gain mechanistic insight into how EMT regulates CMTM6 in breast cancer cells. We used a SNAI1-inducible MCF-7 cell line, where MCF-7 cells were stably transduced with a doxycycline-inducible SNAI1 vector (MCF-7 iSNAI1). We next evaluated whether SNAI1-dependent induction of EMT in epithelial MCF-7 cells could regulate the expression of CMTM6 and impact PD-L1 expression on the surface of cancer cells. Epithelial MCF-7 iSNAI1 cells were treated for five days with increasing doses (0.25–4 µg/mL) of doxycycline (DOX) to determine the appropriate DOX concentration required for the acquisition of mesenchymal features. Our data showed a dose-dependent increase in the expression of SNAI1 with concomitant downregulation of the epithelial marker E-cadherin and upregulation of the mesenchymal marker N-cadherin (Figure 2A). This so-called “cadherin switch” is a typical feature of EMT [21]. Our results revealed that the lower DOX dose producing significant induction of SNAI1 and N-cadherin with marked reduction of E-cadherin proteins was observed using 2 µg/mL DOX (Figure 2A). Furthermore, we showed that, at this DOX concentration (2 µg/mL), the “cadherin switch” was only observed after five days of treatment (Figure 2B). Based on these data, we defined the experimental conditions at 2 µg/mL DOX treatment for 5 days to be used to induce a full EMT switch in MCF-7 iSNAI1 cells. Throughout this study, MCF-7 iSNAI1 cells not treated or treated with DOX were termed as MCF-7^Epi^ (for epithelial) or MCF-7^Mes^ (for mesenchymal), respectively (Figure 2C). Relative to MCF-7^Epi^ cells, we detected mRNA up-regulation of the mesenchymal markers SNAI1, SNAI2, ZEB1, and CDH2, and downregulation of the epithelial marker CDH1 in MCF-7^Mes^ cells (Figure 2D). Confocal microscopy images confirmed the epithelial and mesenchymal phenotypes of MCF-7^Epi^ and MCF-7^Mes^ cells, respectively, characterized by a profound reorganization of the actin cytoskeleton, together with a loss of E-cadherin expression and cell to cell contact in MCF-7^Mes^ cells (Figure 2E). Upon SNAI1-dependent EMT induction, PD-L1 expression was significantly upregulated at both mRNA and protein levels in MCF-7^Mes^ compared with MCF-7^Epi^ cells (Figure 2F). However, under these experimental conditions only CMTM6 protein and not CMTM6 mRNA, was significantly upregulated (Figure 2F and Supplementary Materials Figure S3). Therefore, it is tempting to speculate that EMT-dependent induction of SNAI1 increased CMTM6 protein through yet undefined post-translational mechanism(s).

Previously, a high expression level of CMTM6 was associated with poor prognosis in malignant gliomas [22]. Our data agree with a recent report showing that TGF-β was able to increase CMTM6 expression along with EMT activation in head and neck squamous cell carcinoma (HNSCC) [23].

Next, we evaluated the contribution of SNAI1-dependent induction of CMTM6 in the upregulation of PD-L1 in MCF7^Mes^ cells. We transfected two different siRNAs targeting CMTM6 in MCF-7^Mes^ cells and detected a significant decrease in the expression of CMTM6 mRNA and protein compared with CTRL siRNA transfected cells (Figure 2G, left and middle panels and Supplementary Materials Figure S4). Surprisingly, we observed only a slight (25%) but significant decrease in cell surface expression of PD-L1 in MCF-7^Mes^ cells silenced for CMTM6 (Figure 2G, right panel). Our data indicate that, despite the efficient targeting of CMTM6 expression in MCF-7^Mes^ cells, 75% of the PD-L1 is still present on the cell surface.

It has been recently reported that PD-L1 downregulation, observed in CMTM6-knockdown HNSCC cells, results in significant inhibition of the tumor growth in an allograft mouse model and a reduction in the proportion of PD-1+, TIM-3+, VISTA+, LAG-3+ and B7-H3+ exhausted T cells [23]. Further investigations are required to assess whether the decrease in PD-L1 cell surface expression observed in mesenchymal breast cancer cells targeted for CMTM6 will be able to inhibit tumor growth and mitigate T cell exhaustion in vivo. Nevertheless, our results provide the first evidence of a role of EMT transcription factor SNAI1 in the overexpression of PD-L1 via positive regulation of the CMTM6 expression in breast cancer cells.

### 3.3. CMTM7 Positively Regulates SNAI1-Dependent Expression of PD-L1 in MCF-7 Cells

**Figure 3:**
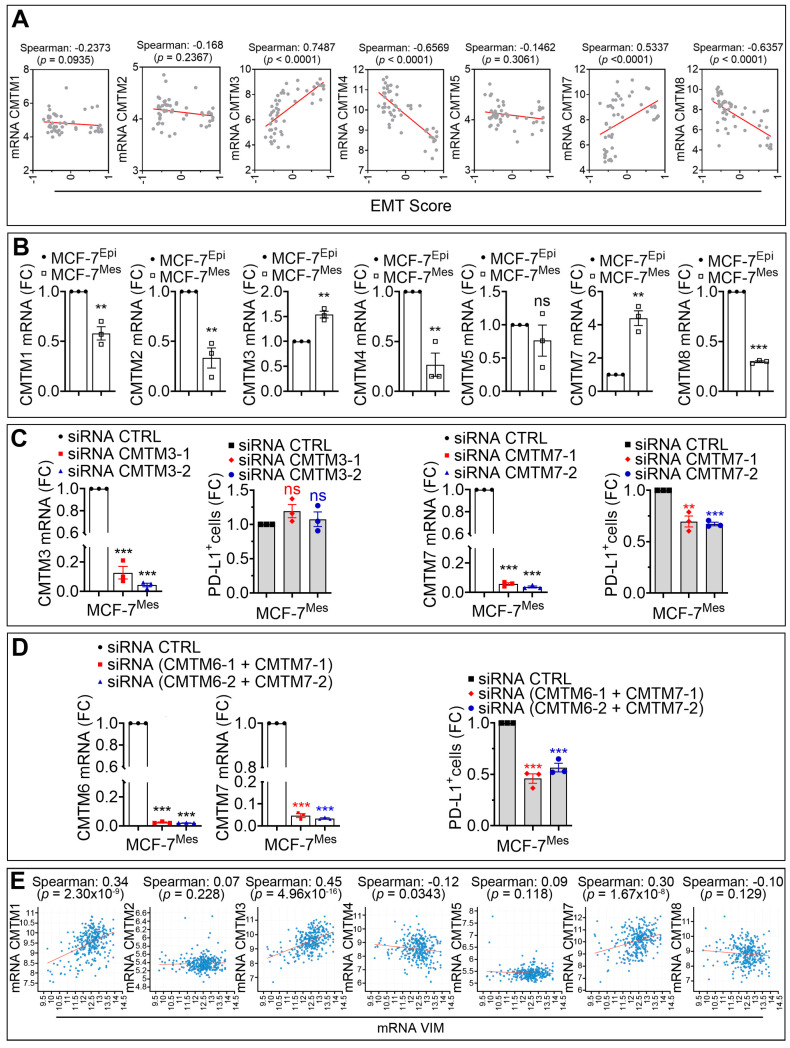
CMTM7 positively regulates the SNAI1-dependent expression of PD-L1 in MCF-7 cells. (**A**) Spearman correlation between EMT score and the mRNA of CMTM1, CMTM2, CMTM3, CMTM4, CMTM5, CMTM7, and CMTM8 in 51 breast cancer cell lines reported in CCLE database. (**B**) Expression of CMTM1, CMTM2, CMTM3, CMTM4, CMTM5, CMTM7, and CMTM8 by mRNA in MCF-7^Epi^ and MCF-7^Mes^ cells. Results are reported as FC compared with MCF-7Epi cells. (**C**) Left panels: expression of CMTM3 mRNA and flow cytometry quantification of PD-L1+ cells in MCF-7^Mes^ cells transfected with CTRL siRNA or two different CMTM3 siRNAs (siCMTM3-1 and -2). Right panels: expression of CMTM7 mRNA and flow cytometry quantification of PD-L1+ cells in MCF7^Mes^ cells transfected with CTRL siRNA or with two different CMTM7 siRNAs (siCMTM7-1 and -2). Results are reported as FC compared with cells transfected with CTRL siRNA. (**D**) Left and middle panels: expression of CMTM6 and CMTM7 mRNA in MCF-7^Mes^ cells transfected with CTRL siRNA or two different combinations of CMTM6 and CMTM7 siRNAs. Right panel: Flow cytometry quantification of PD-L1+ cells. Results are reported as FC relative to cells transfected with CTRL siRNA. Results in (**B**–**D**) represent the average of three independent experiments. Statistically significant differences (indicated by asterisks) are calculated compared to control conditions using an unpaired two-tailed Student’s *t*-test (** *p* < 0.01 and *** *p* < 0.001). (**E**) Correlation between the mRNA expression of VIM and CMTM1, CMTM2, CMTM3, CMTM4, CMTM5, CMTM7, and CMTM8 in TNBC patients reported in METABRIC cohort. The spearman correlation score and the *p*-values are reported. For all panels, each dot represents one individual experiment.

The CMTM family contains eight members CMTM1 to CMTM8 [12]. We next evaluated the involvement of SNAI1 in the regulation of other members of the CMTM family and assessed the mRNA level of CMTM1, 2, 3, 4, 5, 7, and 8 in 51 breast cancer cell lines reported in CCLE database. Our *in silico* data performed on breast cancer cell lines showed that, in addition to CMTM6 (see Figure 1A), EMT score positively correlated with CMTM3 and CMTM7 and negatively correlated with CMTM1, 2, 4, 5, and 8 (Figure 3A). Our experimental results confirm the *in silico* data by demonstrating that the mRNA expression level of CMTM3 and CMTM7 was significantly increased in MCF-7^Mes^ relative to MCF7^Epi^ cells. In contrast, a decrease in the expression of CMTM1, CMTM2, CMTM4, and CMTM8 was observed, while no difference in the expression of CMTM5 was detected (Figure 3B). Recent data have highlighted the role of CMTM3 and CMTM7 in anti-tumor function. Briefly, knocking down CMTM3 promotes metastasis of gastric cancer via the STAT3/Twist1/EMT signaling pathway [24], and knocking down CMTM7 increases tumorigenicity of human non-small cell lung cancer cells [25]. To explore the involvement of EMT-dependent upregulation of CMTM3 and CMTM7 on the expression of PD-L1 in our MCF-7 iSNAI1 model, we targeted either CMTM3 or CMTM7 expression in MCF-7^Mes^ cells by siRNA. Despite the efficiency of siRNA in targeting CMTM3, no impact on the cell surface expression of PD-L1 in MCF-7^Mes^ cells was observed (Figure 3C, left panels). However, CMTM7 silencing induced, similar to CMTM6, a 25% decrease in the cell surface expression of PD-L1 in MCF-7^Mes^ cells (Figure 3C, right panels). Interestingly, the dual knockdown of both CMTM6 and CMTM7 had an additive effect since the decrease in the cell surface expression of PD-L1 reached around 50% (Figure 3D). These data clearly show that, in addition to CMTM6, CMTM7 also contributes to PD-L1 cell surface expression on tumor cells. To our knowledge, these data are the first to demonstrate that the cell surface expression of PD-L1, overexpressed in mesenchymal tumor cells, occurs via both CMTM6 and CMTM7.

We further investigated whether a correlation exists between the major EMT marker VIM and the different members of CMTM in TNBC. Our *in silico* data reported in Figure 3E shows—similar to the results obtained in cell lines—a positive correlation between the expression of Vimentin and both CMTM3 and CMTM7 in TNBC patients.

## 4. Conclusions

Overall, this study provides new insights into the significant role of CMTM6 and CMTM7 in the regulation of PD-L1 surface expression in breast cancer cells undergoing EMT. Having demonstrated the involvement of SNAI1, further studies must be carried out to dissect whether other EMT-TFs, such as SNAI2, ZEB1, or TWIST, are also involved in the regulation of the CMTM members in breast cancer cells. In addition, further investigations should be done to identify potential (i) post translational modification involved in CMTM6 protein regulation, (ii) motifs in the promoter of CMTM3 and CMTM7 family members that could bind EMT-TFs. Furthermore, we believe that the functional impact of PD-L1 regulation via CMTM6 and CMTM7 on tumor cell susceptibility to CTL-mediated killing needs to be investigated in vitro and in vivo.

Despite the exciting and encouraging clinical responses in diverse malignancies, anti-PD-1/PD-L1-based immunotherapy failed to achieve high objective responses, and yielded modest response rates in metastatic breast cancer. This study provided mechanistic evidence about the role of EMT in the regulation of PD-L1 via CMTM6 and CMTM7. We strongly believe that such mechanistic understanding will provide a framework for the development of assay aiming to simultaneously investigate a correlation between the expression of PD-L1 and CMTM6/7 in clinical samples. Nevertheless, our data presented here will pave the way towards the development of innovative treatments aimed at regulating the expression of PD-L1 via modulating CMTM6 and CMTM7 proteins in tumors highly addicted to the EMT process. In metastatic breast cancer patients, EMT inhibitors can be combined with PD-L1 blockade to improve their response rates.

## Data Availability

Publicly available datasets of TCGA and CCLE were analyzed in this study.

## Supplementary Materials

The following are available online at https://www.mdpi.com/2072-6694/13/5/1165/s1. Figures S1–S4: Densitometry quantification of Western blot of Figure 1F(1) and G(2), Figure 2F(3) and G(4). Table S1: List of primers used.
