# Supplementary material for: Epithelial to Mesenchymal Transition Regulates Surface PD-L1 via CMTM6 and CMTM7 Induction in Breast Cancer
Source: Cancers (Basel). 2021 Mar 9;13(5):1165. doi: 10.3390/cancers13051165 (PMC7963182; doi:10.3390/cancers13051165)
Supplement: Supplementary file 1 [file cancers-13-01165-s001.pdf]

cancers

MDPI

Supplementary Materials: The following are available online at https://www.mdpi.com/2072-6694/13/5/1165/s1.

Table S1. List of primers used.

Primer Name	Primer Sequence
CMTM1-F	GGCCATGCAAGAAAAGAAAA
CMTM1-R	CGATGCAACACACGATTACC
CMTM2-F	CGGCTGGAGTTTTTGCTTT
CMTM2-R	CTGGGGTCCTTTTTCCTTTC
CMTM3-F	GGCTTTCCTCTGCTCTCTCA
CMTM3-R	GAGGACGCCACATAGCAGAT
CMTM5-F	TGTTTCTCTTGCTGGGCTTT
CMTM5-R	CCTGGAGCTCTGCAACTACC
CMTM7-F	TGATCCTCGCCTTTTACCTG
CMTM7-R	GAGGAGCAGGGTACCGATTA
CMTM8-F	CCTGAGAGGGACAGTCACAAC
CMTM8-R	ATTTCCAGCGTAGCAGATGG
SNAI1-F	CGAAAGGCCTTCAACTGCAAA
SNAI1-R	TGACATCTGAGTGGGTCTGGA
SLUG-F	CTACAGCGAACTGGACACACA
SLUG-R	TGGAATGGAGCAGCGGTAGT
ZEB1-F	CCTCTTCAGGTGCCTCAGGAAAA
ZEB1-R	CCTCTTCAGGTGCCTCAGGAAAA
VIMENTIN-F	CCTTGAACGCAAAGTGGAATC
VIMENTIN-R	GACATGCTGTTCCTGAATCTGAG
CDH1-F	AGTGCCAACTGGACCATTCA
CDH1-R	TCTTTGACCACCGCTTCTCT
CDH2-F	CTGCGCTGTAAACATCTTCAG
CDH2-R	CTCCATGTGCCGGATAGC
PDL1-F	TGCCGACTACAAGCGAATTACTG
PDL1-R	CTGCTTGTCCAGATGACTTCGG
ACTIN-F	GGTGGCTTTTAGGATGGCAAG
ACTIN-R	ACTGGAACGGTGAAGGTGACAG

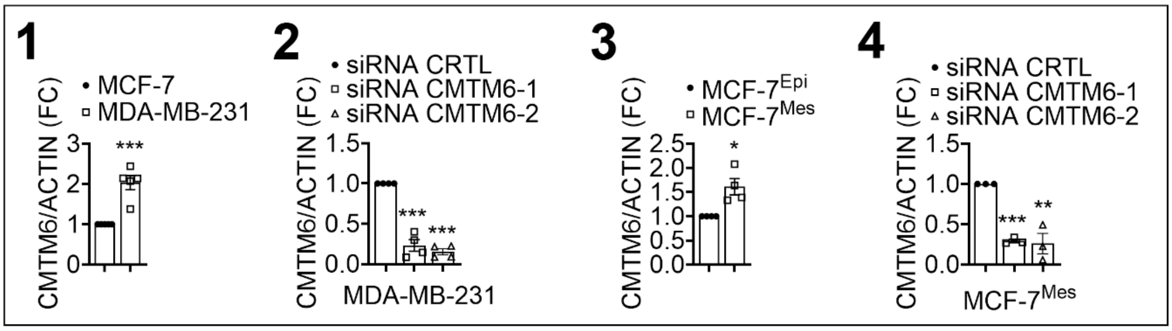

Figure S1-4. Densitometry quantification of Western blot of Figures 1F (1), 1G (2), 2F (3) and 2G (4). The quantification of CMTM6 protein expression was done relative to actin and reported as fold change compared to the control condition (considered as 1). Each dot represents one experiment. Statistically significant differences +/- SEM (indicated by asterisks) are calculated using an unpaired two-tailed Student's t-test (* $p < 0.05$; ** $p < 0.01$ and *** $p < 0.001$).
